# Letter to the Editor: Bean-Associated Cytorhabdovirus and Papaya Cytorhabdovirus are Strains of the Same Virus

**DOI:** 10.3390/v11030230

**Published:** 2019-03-07

**Authors:** Nicolás Bejerman, Ralf G. Dietzgen

**Affiliations:** 1 UFyMA-INTA-Conicet, Córdoba 5020, Argentina; 2 Queensland Alliance for Agriculture and Food Innovation, The University of Queensland, St. Lucia, QLD 4072, Australia

**Keywords:** bean-associated cytorhabdovirus, papaya cytorhabdovirus, taxonomy, cytorhabdovirus

## Dear Editor,

**Figure 1:**
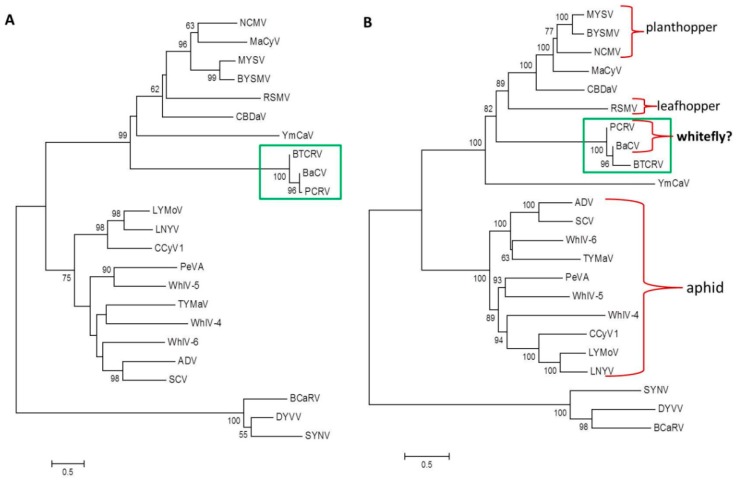
Neighbor joining trees inferred using an alignment of nucleocapsid (N protein) (**A**) and RdRp (L protein) (**B**) from 20 available cytorhabdovirus genome sequences. The genomes of the nucleorhabdoviruses sonchus yellow net virus (SYNV; L32603), datura yellow vein virus (DYVV; KM823531) and *black currant-associated rhabdovirus* (BCaRV; MF543022) were used as outgroup. The trees were constructed using the JTT model with 1000 bootstrap replicates. BaCV, PCRV, and BTCRV are within a green box. (**B**) also indicates known insect vector types. The cytorhabdoviruses used to construct the tree, and their accession numbers, are: alfalfa dwarf virus (ADV; KP205452), barley yellow striate mosaic virus (BYSMV; KM213865), bean-associated cytorhabdovirus (BaCV; MK202584) *Bemisia tabaci* virus-like sequences (BTCRV; KJ994260; KJ994261; KJ994263; KJ994264), cabbage cytorhabdovirus-1 (CCyV-1; KY810772), colocasia bobone-disease associated virus (CBDaV; KT381973), lettuce necrotic yellow virus (LNYV; AJ867584), lettuce yellow mottle virus (LYMoV; EF687738), maize-associated cytorhabdovirus (MaCyV; KY965147), maize yellow striate virus (MYSV; KY884672), northern cereal mosaic virus (NCMV; AB030277), papaya cytorhabdovirus (PCRV; MH282832), persimmon virus A (PeVA; AB735628), rice stripe mosaic virus (RSMV; KX525586), strawberry crinkle virus (SCV; MH129615), tomato yellow mottle-associated virus (TYMaV; KY075646), wuhan insect virus 4 (WhIV-4; KM817650), wuhan insect virus 5 (WhIV-5; KM817651), wuhan insect virus 6 (WhIV-6; KM817652), yerba mate chlorosis-associated virus (YmCaV; KY366322).

Recently, Alves-Freitas and colleagues [1] reported the complete genome sequence of a new cytorhabdovirus associated with a common bean in Brazil (GenBank accession number MK202584) which they tentatively named bean-associated cytorhabdovirus (BaCV). These authors reported that there was a low level of sequence identity with other cytorhabdoviruses (15-39%) and that RNA-dependent RNA polymerase (RdRp) phylogeny showed that the BaCV clustered most closely with yerba mate chlorosis-associated virus (YmCaV) and rice stripe mosaic virus (RSMV). Based on these results, the authors suggested that BaCV should be taxonomically classified as a new species in the genus *Cytorhabdovirus.* However, when we subjected BaCV protein sequences to Blastp analysis, the top hit (organism) was papaya cytorhabdovirus (PCRV) (GenBank accession number MH282832), whose complete genome sequence has been available in GenBank since October 1, 2018, almost three months before the manuscript describing BaCV was submitted to *Viruses*; the authors of the BaCV study may have overlooked the GenBank submission, since the work describing papaya cytorhabdovirus has not yet been published. This finding prompted us to further investigate the relationship between BaCV and PCRV. The genomic organization of both viruses, as well as the gene junction sequences are very similar. Moreover, when analogous genes of both viruses were compared to each other, the sequence identity was always above 92%, and as high as 96%, for the nucleocapsid protein (N) gene, and 97% for glycoprotein (G) and RdRp genes/proteins (Table 1), which suggests that BaCV and PCRV are strains of the same virus. Viruses assigned to different species within the genus *Cytorhabdovirus* have a minimum nucleotide sequence divergence of >50% in cognate genes [2], which is clearly not the case here. Interestingly, both viruses have a high amino acid sequence identity (over 89%) with virus-like sequences associated with the whitefly *Bemisia tabaci* available in GenBank (KJ994257; KJ994260; KJ994261; KJ994263; KJ994264 accession numbers). Phylogenetic analysis showed that the N (Figure 1A) and RdRp (Figure 1B) amino acid sequences of BaCV, PCRV, and *Bemisia tabaci* virus-like sequences (BTCRV) cluster together in a separate clade, indicating that these viruses are closely related. This phenomenon indicates that BaCV and PCRV are strains of the same virus and that *Bemisia tabaci* is a likely potential vector of these viruses (Figure 1B). 

In conclusion, considering all the available data, the bean-associated cytorhabdovirus identified by Alvares-Freitas and collaborators [1] should be correctly classified as a bean-infecting strain of papaya cytorhabdovirus and not as a new virus. Our sequence analysis further suggests that this virus may be the first rhabdovirus to be potentially transmitted by whiteflies.

## Figures and Tables

**Table 1:** Sequence identities between bean-associated cytorhabdovirus and papaya cytorhabdovirus.

	identity % (nt/aa)
genome	96.5
3’leader	93.8
N	96.8/96.9
P	97.0/94.8
P3	96.4/97.3
P4	94.1/92.4
M	96.6/98.6
G	97.3/97.9
L	97.3/97.9
5’trailer	95.2

nt: nucleotides; aa: amino acids.
